# Identification of Cyclopropane Fatty Acids in Human Plasma after Controlled Dietary Intake of Specific Foods

**DOI:** 10.3390/nu12113347

**Published:** 2020-10-30

**Authors:** Veronica Lolli, Margherita Dall’Asta, Daniele Del Rio, Augusta Caligiani

**Affiliations:** 1 Department of Food and Drug, University of Parma, 43124 Parma, Italy; veronica.lolli@unipr.it (V.L.); augusta.caligiani@unipr.it (A.C.); 2 Department of Animal Science, Food and Nutrition, Università Cattolica del Sacro Cuore, 29122 Piacenza, Italy; 3 Department of Veterinary Science, University of Parma, 43126 Parma, Italy; daniele.delrio@unipr.it; 4 School of Advanced Studies on Food and Nutrition, University of Parma, 43124 Parma, Italy

**Keywords:** dihydrosterculic acid, food intake, dairy foods, Grana Padano cheese, GC-MS, in vivo bioaccessibility

## Abstract

Cyclopropane fatty acids (CPFAs) are an investigated class of secondary fatty acids of microbial origin recently identified in foods. Even though the dietary daily intake of this class of compounds it has been recently estimated as not negligible, to date, no studies specifically have investigated their presence in human plasma after consumption of CPFA-rich sources. Therefore, the aims of this study were (i) to test CPFAs concentration in human plasma, thus demonstrating their in vivo bioaccessibility and potential bioavailability, (ii) to investigate a dose-response relationship between medium term chronic intake of CPFAs-rich foods and both CPFAs and plasma total fatty acid profiles in healthy subjects. Ten healthy normal weight adults were enrolled for conducting an in vivo study. Participants were asked to follow a CPFA-controlled diet for 3 weeks, consuming 50 g of Grana Padano cheese (GP) and 250 mL of whole cow milk, which correspond to a total of 22.1 mg of CPFAs. Fasting CPFAs concentration were monitored for eight timepoints during the whole study and plasma total fatty acids composition was determined by GC-MS. CPFAs, mainly dihydrosterculic acid (DHSA), were identified in plasma total fatty acids profile at the beginning of the study and after dietary treatment. A significant (*p* < 0.05) increase of CPFAs mean plasma concentration (*n* = 10) were observed at the end of the dietary intervention. Contrarily, the total fatty acids composition of the general plasma fatty acids profile did not significantly change (*p* ≥ 0.05) during the dietary intervention period. This is the first investigation demonstrating that CPFAs are bioaccessible in vivo and, as expected, their plasmatic concentration may be affected by consumption of CPFAs-rich foods. This research will open the door to further detailed research, which may better elucidate the role of these compounds in human health.

## 1. Introduction

Cyclopropane fatty acids (CPFAs), as dihydrosterculic (cis-9,10-methyleneoctadecanoic) (DHSA) and lactobacillic (cis-11,12-methyleneoctadecanoic) acids, are unusual alicyclic fatty acids that occur in plants, fungi, and both Gram-negative (e.g., Escherichia coli) and Gram-positive (e.g., Lactobacillus sp.) microorganisms, as well as Protozoa and Myriapoda [1,2,3,4]. Some papers suggest that CPFAs are involved in the bacterial pathogenesis of infections and in the resistance of some bacterial strains such as Lactobacillus sp., Escherichia coli, Salmonella enterica, Staphylococcus aureus, and Pseudomonas to different environmental stresses (e.g., temperature changes, high osmolarity, solvents, acid pH and others), as the presence of antibiotics or heavy metals in the culture medium [5,6]. However, the real significance of these fatty acids in their natural context is poorly known, as well as their occurrence in higher animals. Recently, CPFAs were detected in significant amounts, especially in cow milk and several dairy products (ranging between 9.0–30.0 mg/100 g) and in bovine meat (ranging between 0.7–4.0 mg/100 g) but also in some species of fish and mushrooms [7,8,9,10]. The occurrence of CPFAs in foods has been suggested to mainly derive from the use of silages in animal feeding, where CPFAs can be released by epiphytic lactic acid bacteria (LAB) during the ensiling process [7]. All these previous data indicated that the environmental conditions developed in maize silos are essential for the production and release of CPFAs from LAB in cow milk, whereas rumen digestion and food processing did not affect CPFAs content [7,8,10]. Our previous results [10] also showed that the most important CPFAs food sources in the Italian diet were dairy products and bovine meat. CPFAs daily intake from these food items was not negligible, and worth studying to investigate their potential biological effects on humans. Furthermore, we recently reported CPFAs digestibility after in vitro simulated human gastrointestinal digestion [11], so their potential bioaccessibility and distribution in human tissues. Existing human studies identified CPFAs (mainly cyclopropaneoctanoic acid 2-hexyl) in both human serum and adipose tissue [12,13], suggesting that these fatty acids are efficiently absorbed and may play a physiological role in the human body. Moreover, fatty acids containing cyclopropenoid rings (especially the unsaturated analogues) have been reported to influence lipid metabolism, kidney function, inflammation, and enzymes activity as cyclooxygenase and stearoyl-CoA desaturase [14,15,16,17,18,19]. To the best of our knowledge, no information is present in the literature about the fate of CPFAs within the human body, and a thorough investigation on CPFAs in vivo bioaccessibility and how they accumulate and are metabolized in humans is needed. Therefore, any data about their occurrence in vivo are particularly useful, to give a further understanding of the interrelationship between diet, bioaccessibility, and human health. This work deals with a pilot investigation to determine CPFAs in human plasma after the administration of tested CPFA-containing foods, in order to assess their plasmatic concentration, proving their presence in human plasma, and possibly to correlate their presence with the medium-term chronic exposure to CPFAs-rich foods.

## 2. Materials and Methods

### 2.1. Ethics

The study was conducted according to the guidelines of the Declaration of Helsinki and the research protocol was approved by IRB—Institutional Review Board of the University of Parma (Prot. n. 0081/2017) and registered on www.clinicaltrial.gov (ID: NCT03612700) before the starting. All subjects gave written informed consent to participate in the study.

### 2.2. Participant Characteristics

A total of ten (*n* = 10) healthy subjects were recruited and they attended a screening visit to determine eligibility for the study. The exclusion criteria were age (<18), BMI < 19 kg/m^2^ or ≥30 kg/m^2^, any gastrointestinal diseases (e.g., irritable bowel syndrome, infections, diverticular diseases, colitis, colon polyps, autoimmune intestinal diseases and cancer), coeliac disease, hypertriglyceridemia (>150 mg/dL) and hypercholesterolemia (>200 mg/dL), use of food supplements or medicaments interfering with lipid metabolism, pregnancy, and breastfeeding.

Characteristics of the subjects enrolled at the baseline are presented in Table 1.

### 2.3. CPFAs-Rich Foods

GP (24 months of ripening) and whole UHT cow milk (3.5% of fat) of the same brand from the same batch were purchased from the same Italian retailer and provided to each participant for the entire duration of the study. Before the study, the CPFAs content of these selected foods was tested following the previously validated method published by Caligiani et al. 2016 [20]. CPFAs concentrations ranged between 100.4 and 110.5 mg/100 g total fat for cheese, and 77.8 and 84.4 mg/100 g total fat for whole milk, in accordance with the previous data [20]. The nutritional values and CPFAs mean content per portion consumed by each participant are reported in Table 2.

### 2.4. Dietary Intervention

Prior to the dietary intervention, each subject was asked to refrain any CPFAs source for 1 week in order to avoid any confounding factors. After 1 week of washout, each participant was asked to follow a free-living diet, with a controlled CPFAs intake. A list of permitted and forbidden foods was supplied to the participants. Additionally, the compliance to the recommended diets was monitored. Subjects were asked to daily report the consumption of foods included in the “permitted” or “forbidden” lists, in addition to any changes that occurred during the study, for monitoring the exact amount of CPFAs ingested both during the 1-week of restricted diet and the 3-week CPFA-controlled intake. The CPFAs controlled daily intake consisted of 15.3 ± 1.0 mg of CPFAs from 50 g of GP and 6.8 ± 0.4 mg of CPFAs from 250 mL of whole UHT cow milk, for a total of about 22.1 mg of CPFAs/day.

**Figure 1:**
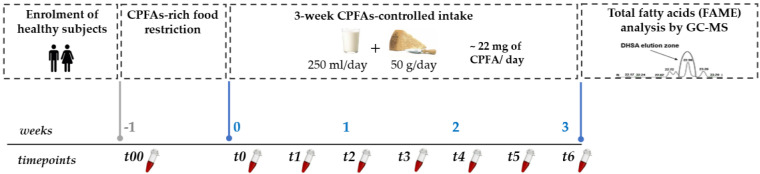
Graphical representation of the study design. CPFAs: cyclopropane fatty acids; DHSA: dihystrosterculic acid; FAME: fatty acids methylesters.

Capillary whole blood of each volunteer was collected by finger-prick at fasting state at eight timepoints during the study: (a) t00 (baseline): after the enrolment; (b) t0: after a 1-week washout period (dairy products and bovine meat restricted diet); (c) t1, t2, t3, t4, t5, t6: twice a week during the dietary treatment under a free-living diet with a controlled CPFAs intake for three weeks. All capillary blood samples were collected in heparin fluoride tubes, centrifuged for 2 min at 10,000 rpm (Centrisart^®^ A-14 Micro-Centrifuge, Sartorius GmbH, Göttingen, Germany). Finally, the upper plasma layer was withdrawn and divided into two aliquots (240 µL), dried under vacuum and stored at −80 °C until fatty acids analysis. The study design is shown in Figure 1.

### 2.5. Post-Prandial CPFAs Response

Prior to the intervention study, one subject was asked to perform an acute pilot post-prandial study for investigating the CPFAs blood response curve after consumption of a portion of GP cheese. Following a standard diet for 24 h without any source of CPFAs-rich foods, the volunteer was asked to consume at fasting state either a portion (160 g) of GP with one package of cracker and a cup of coffee or the same meal without any CPFA-rich source (control meal). Capillary blood was collected, applying the same method described for chronic study, at fasting state and after 1 h, 2 h, 3 h, 4 h, 6 h, 8 h, and 24 h after GP intake.

### 2.6. Plasma Sample Preparation

Total fatty acids methyl esters (FAME) were obtained from plasma samples according to the protocol reported by Han et al. (2011) [22] with some modifications. Each aliquot of dried plasma (240 µL) was treated with 1 mL of 5% HCl/CH_3_OH at 70 °C for 30 min. After cooling, 200 µL of distilled water and 5 µL of tetracosane (Sigma Aldrich, St. Louis, MO, USA; purity 99%) standard solution at 100 µg/g, as internal standard, were added. Finally, FAME were extracted twice with 1 mL of hexane, the final aliquots collected and evaporated to dryness in a stream of nitrogen. One sample of FAME extract (at t00) was spiked with DHSA pure standard methylester (Abcam, Cambridge, UK, purity >98%) as reference material for CPFAs identification.

### 2.7. GC-MS Analysis

GC-MS analysis of FAME was performed following the conditions in reference to Caligiani et al. (2016) [20], slightly adapted. FAME (1 µL, split mode 20:1) were analyzed with a Thermo Scientific Trace 1300 gas-chromatograph coupled to a Thermo Scientific Trace ISQ mass spectrometer (Thermo Scientific, Waltham, Massachusetts, USA). A low-polarity capillary column (SLB-5ms, 30 m × 0.25 mm, 0.25 µm of thickness, Supelco, Bellafonte, PA, USA) was used. The chromatogram was recorded in the sim/scan mode (SCAN: 40–500 *m*/*z*; SIM: 55, 74, 87, 278, 282, 270, 298, 310 *m*/*z*) with a programmed temperature from 60 °C to 280 °C. The initial oven temperature was 60 °C, held for 2 min, subsequently increased to 220 °C at a rate of 10 °C min^−1^, maintained for 8 min, increased to 280 °C at a rate of 20 °C min^−1^ and held isothermal for 17 min. The ion source temperature was set at 230 °C. Peak identification of CPFAs was performed based on the mass spectrum previously published (characteristic ions for lactobacillic and dihydrosterculic methylesters m/z 278; M ^+^: *m*/*z* 310) [7] and on the reference library (NIST 11), then compared with the mass spectrum and retention time of DHSA pure standard methylester in the calibration solution [20]. For further certainty, the identification of CPFAs signal was supported by the spike of a sample of FAME extract from plasma (see above) by DHSA standard methylester. Quantification was performed as described by Caligiani et al. [20], considering the area ratio between the analyte and the internal standard tetracosane, and volume of plasma used for the procedure. Finally, CPFAs concentration was expressed as µmol/L. In addition, three timepoints were chosen to compare the effect of the three dietary patterns: (i) before dietary treatment (t00 as baseline); (ii) wash out (t0); (iii) dietary treatment end (t6), on the general fatty acid composition of human plasma expressed as relative percentage mean of total lipids of all subjects.

### 2.8. Statistical Analysis

No previous study specifically investigated the bioavailability of DHSA and lactobacillic acids in humans. Therefore, samples size of this pilot study was fixed according to the number of subjects normally included for studies investigating bioavailability of dietary compounds [23,24]. Statistical analyses were performed utilizing SPSS statistical software (Version 25.0, SPSS Inc., Chicago, Illinois, USA) and differences were considered significant at *p* < 0.05. All data collected were assessed for normality using the Kolmogorov-Smirnov test, and the normal distribution was confirmed. All results are reported as mean values ± standard error of the means (SEMs), unless otherwise stated. Comparison between CPFAs mean concentration after washout (t0) and at each timepoint during the dietary treatment (t1; t2; t3; t4; t5; t6) was performed using the paired samples T-test. One-way analysis of variance (ANOVA) (with repeated measures and Bonferroni post-hoc) was applied on the general fatty acid plasma profile at three selected time points (t00, t0 and t6).

## 3. Results

### 3.1. CPFAs Detection and Quantification in Human Plasma

**Figure 2:**
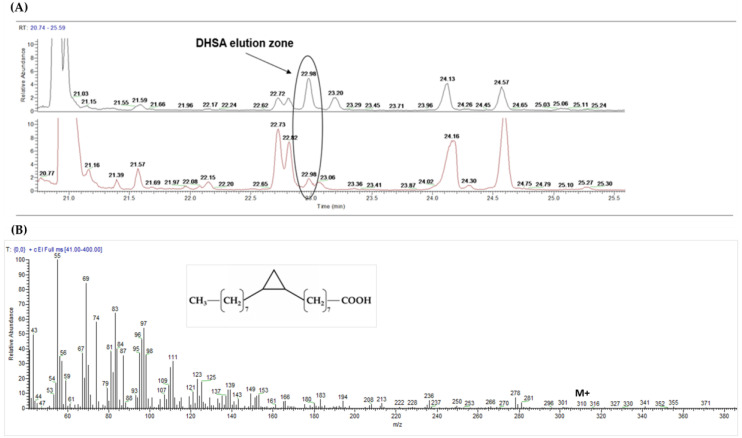
GC-MS detection of cyclopropane fatty acids in human plasma. DHSA: dihydrosterculic acid. (**A**) zoom of the elution zone (retention time: 22.98 min) of DHSA methyl ester in spiked plasma fat at t00 (black) compared with that detected in human plasma of one subject at t6 (red); (**B**) DHSA chemical structure and mass spectrum detected in human plasma sample at t6 (full SCAN mode; characteristic ion: *m*/*z* 278; M +: *m*/*z* 310).

CPFAs, have been detected and quantified by GC-MS according to Caligiani et al. (2016) [20] as the sum of two isomers (DHSA and lactobacillic acids) and as minor components in the total plasma fatty acid profile of all volunteers and at each timepoint. The identification of CPFAs signal (see Figure 2) was supported by the retention time and the full SCAN mass spectrum of CPFAs signal detected in human plasma samples, that are identical to those detected in the spiked sample of the FAME extract from plasma by DHSA pure standard methyl ester (the major isomer).

### 3.2. Chronic Study

A total of ten (*n* = 10) healthy subjects (6 females) started and concluded the study without any violation of the protocol. All participants consumed all the provided meals (GP and whole UHT cow milk) daily for 3 weeks, according to the study protocol and plasma samples were analyzed for CPFAs and lipid profile. The body weight was monitored throughout the study for verifying potential changes during the treatment, and no significant changes (one way ANOVA, *p* ≥ 0.05) were observed (change of body weight at t6 compared to t00 was 1.1 ± 2.4 kg).

**Figure 3:**
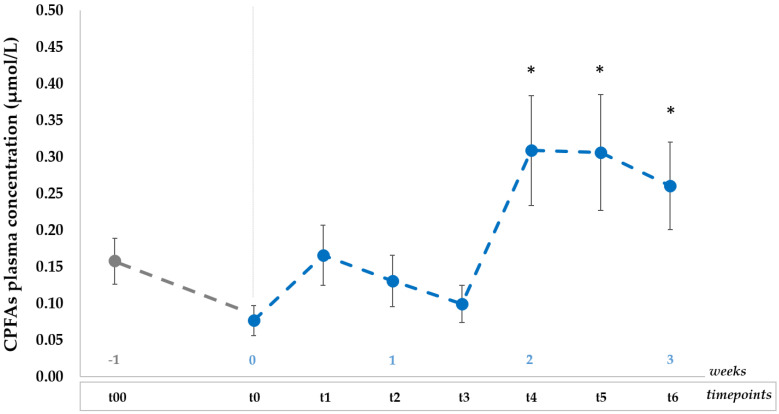
Plasma CPFAs mean concentrations (µmol/L, mean ± SEMs) over time for all the subjects (*n* = 10) consuming daily portions of GP and whole cow milk for three weeks after 1 week of restricted CPFAs diet (baseline t00 and washout t0); data are expressed as mean ± SEM; * *p* < 0.05 after paired samples T-test between each timepoint (t1, t2, t3, t4, t5, t6) vs. t0. CPFAs: cyclopropane fatty acids; it refers to the sum of two isomers, namely dihydrosterculic acid (DHSA) and lactobacillic acid [20].

Results of CPFAs mean plasmatic concentrations (*n* = 10) at baseline (t00), after one-week wash out (t0) and during the 3-week dietary intervention (t1, t2, t3, t4, t5, t6) are shown in Figure 3.

Data indicate there was a relatively large inter-subject variation (% CV) in plasma CPFAs levels, falling in the range between 60–90% at each timepoint.

After one-week of diet with low CPFAs intake, the plasma CPFAs mean concentration decreased (t0, 0.08 ± 0.02 µmol/L) but was not significantly different (*p* ≥ 0.05) from the basal level (t00, 0.16 ± 0.03 µmol/L). On the contrary, significant changes (*p* < 0.05) were observed after CPFAs controlled intake when compared to that measured after the washout (t0), peaking at t4 (0.31 ± 0.08 µmol/L) and at t5 (0.31 ± 0.08 µmol/L) after two weeks of dietary treatment. Moreover, CPFAs plasma level was significantly higher (*p* < 0.05) at last timepoint (t6, 0.26 ± 0.06 µmol/L) than the concentration measured at t0 (washout).

### 3.3. Acute Study

A very preliminary pilot in vivo acute test was conducted with one volunteer, in order to evaluate a CPFAs response curve after a single dose of GP providing 50 mg of CPFAs compared to control (no treatment). As expected, results showed an increase of CPFAs concentration after 4 h for the test meal but not in the control meal, reaching the maximum concentration after 8 h (11.34 ± 0.02 µmol/L, +11.22 µmol/L compared to the starting concentration at fasting state), and turning back to the baseline level (0.09 ± 0.01 µmol/L) after 24 h as shown in Table 3.

### 3.4. Effect of the Treatment on Total Fatty Acids Composition in Human Plasma

The total fatty acids composition of the general plasma lipid profile resulted comparable in all subjects prior to the study and did not change significantly (*p* ≥ 0.05) during the dietary intervention period, except for CPFAs concentration (*p* < 0.05), as previously described. Table 4 reports the amounts of the main fatty acids (FA) determined at basal level (t00), after one-week restricted CPFAs diet (t0) and at the end of the dietary intervention (t6) and expressed as a relative percentage (%) ± SD of the total FA.

## 4. Discussion

In the present pilot in vivo study, GC-MS analysis was carried out to characterize the total fatty acid profile of human plasma focusing on the determination of CPFAs levels before and after a medium-term consumption of CPFAs-rich foods, especially dairy products.

To the best of our knowledge, no previous intervention studies have been specifically conducted for investigating DHSA bioaccessibility in humans after consumption of DHSA-rich foods, and no proper methodologies were available to accurately detect and quantify this marker in human plasma. To date, previous findings [12,13] reported the presence of cyclopropaneoctanoic acid 2-hexyl, namely cis-9,10-methylenhexadecanoic (two carbon shorter chain than DHSA), both in the adipose tissue (stored as triglycerides) and in serum of obese women, but DHSA was not identified because its concentration was lower than their analytical limit of detection. They also suggested that serum levels of that CPFAs were affected by food intake in rats and positively related to high serum triglyceride concentrations observed in obese patients with chronic kidney disease. However, no data about the influence of CPFAs on cellular metabolism in humans were reported. The catabolism of CPFAs in mammals was investigated by Wood and Reiser (1965) [1], who synthesized cis- and trans-9,10-methyleneoctadecanoate and fed weanling rats these molecules for five weeks. The presence of cis- and trans-3,4-methylene dodecanoic acids in rat adipose tissue suggested the inability of the β-oxidation enzyme system to proceed past the cyclopropane ring. In this regard, it cannot be excluded the presence of other plasmatic CPFAs metabolites, related to DHSA, that have not been identified in our experimental conditions. The present pilot study suggested that mainly DHSA, deriving from dairy products, is present in human plasma of healthy normal weight subjects, thus being potentially bioaccessible, and its plasmatic concentration is positively associated with the consumption of CPFAs-rich foods within the diet. Additionally, DHSA was detected in the plasma of all ten volunteers before the dietary intervention, demonstrating that the exposure to CPFAs is quite common and a detectable level is expected to be observed at baseline in the population. Furthermore, these results are supported by preliminary data obtained in the acute study on one subject, which certainly need to be further explored in the future. DHSA was detected after the ingestion of a single dose of Grana Padano cheese, peaking at 8 h (+11.22 µmol/L compared to the starting concentration at fasting state) and going back to baseline level at 24 h. Certainly, it must be pointed out that some population (i.e., in this case from the Northern Italy) might be more exposed to CPFAs than others, in relation to their dietary habits (as a high consumption of dairy foods), in particular on the intake of CPFA-rich sources. Finally, CPFAs have been reported to be produced by specific intestinal bacteria as *Lactobacillus reuteri* [14], making it possible to observe a baseline level of these compounds due to their production and subsequent absorption at colonic level. Different microbiota, besides different dietary habits, may then obviously explain part of the observed variability at t00 in our study. CPFAs are minor fatty acids in the framework of the human plasmatic lipid profile representing around 0.03% of the total FA. However, their amount is comparable to that of other secondary FA, largely studied for their biological effects and putative benefits for human health, like conjugated isomers of α-linolenic acid [25,26]. In this regard, and with rising attention to the health-related effects of dairy lipids, the association of CPFAs with factors linked to human health and disease is becoming paramount. To properly address this matter, we hope that our pilot study may pave the way for bigger and more detailed interventions aimed at understanding the absorption, metabolism, and perhaps the microbiota contribution to the CPFAs circulating panel. Surely, the need remains to systematically investigate CPFAs bioavailability, including in which chemical form they are present in the blood stream and factors influencing their absorption (e.g., dietary components, food formulation).

## 5. Conclusions

The present pilot in vivo investigation described the presence of CPFAs, mainly DHSA, as minor fatty acids in human plasma (0.03 ± 0.01% on total fatty acids) and suggested that their plasmatic levels were dose-dependent: DHSA concentration decreased by 50% after the washout, whereas significantly increased (*p* < 0.05) by about 30% at the end of the 3-week dietary intervention with cow whole milk and Grana Padano cheese. Finally, besides the differences detected for CPFAs, the treatment did not significantly affect (*p* ≥ 0.05) the plasma general fatty acids profile when compared with the profile after one week of CPFAs refrain (washout). Therefore, the results obtained prove that DHSA plasmatic concentration can be strongly affected by the dietary intake of foods containing CPFAs, such as dairy products. The potential biological role of CPFAs within the human body is still unknown. Therefore, future studies should focus on their bioaccumulation in tissues and metabolism in order to establish their role in human health.

## Figures and Tables

**Table 1:** Characteristics of study participants (*n* = 10).

Characteristics of Subjects	Mean ± SD
Age, years	29 ± 6
Weight, kg	69 ± 15
BMI, kg/m^2^	23.4 ± 3.0

SD: Standard Deviation; BMI: body mass index.

**Table 2:** Nutritional values and cyclopropane fatty acids (CPFAs) content (mean ± SD) per portion of CPFAs-rich foods supplied to the volunteers.

	Grana Padano (50 g) ^2^	Whole UHT ^3^ Cow Milk (250 mL) ^2^
Energy (kcal)	196	155
Fats (g)	14.5	8.8
Carbohydrates (g)	0.0	11.6
Fiber (g)	0.0	0.0
Proteins (g)	16.5	8.3
Cyclopropane fatty acids (mg) ^1^	15.3 ± 1.0	6.8 ± 0.4

^1^ Cyclopropane fatty acids refer to the sum of two isomers, namely dihydrosterculic acid (DHSA) and lactobacillic acid, calculated as previously reported by Caligiani et al. (2016) [20], and expressed as mean ± SD of duplicates from two independent extractions; ^2^ Portion size fixed according to the Italian Nutrition Society recommendations for milk and cheese [21]; ^3^ UHT: Ultra-High Temperature.

**Table 3:** CPFAs ^1^ concentrations in human plasma after a test meal (a single dose of GP) and control meal (without CPFAs).

	Plasma CPFAs ^1^ (µmol/L)	
Time (h)	Test Meal ^2^	Control Meal ^3^
0	0.12 ± 0.01	0.12 ± 0.01
1	0.12 ± 0.01	0.12 ± 0.01
2	0.07 ± 0.01	0.12 ± 0.01
3	0.15 ± 0.01	0.12 ± 0.01
4	0.23 ± 0.01	0.12 ± 0.01
6	4.04 ± 0.01	0.12 ± 0.01
8	11.34 ± 0.02	0.12 ± 0.01
24	0.09 ± 0.01	0.12 ± 0.01

^1^ CPFAs: cyclopropane fatty acids; it refers to the sum of two isomers, namely dihydrosterculic acid (DHSA) and lactobacillic acid [20]; ^2^ Test Meal = breakfast with single dose (160 g) of GP (providing 50 mg of CPFAs); ^3^ Control Meal = breakfast without any CPFAs sources. Values are expressed as means ± SEMs of two duplicates.

**Table 4:** General fatty acids composition (g/100 g) of total fatty acids methyl esters (FAME) detected in human plasma at the selected timepoints (t00, t0, t6).

		g/100 g Total FAME	
FAME	t00	t0	t6
C12:0 Lauric	0.53 ± 0.11	0.32 ± 0.07	0.40 ± 0.08
C14:0 Myristic	3.18 ± 0.65	2.10 ± 0.37	2.62 ± 0.51
C16:0 Palmitic	16.13 ± 0.70	24.33 ± 1.16	14.68 ± 0.64
C18:0 Stearic	11.36 ± 1.72	9.21 ± 1.60	9.87 ± 1.48
Other SFA	3.76 ± 0.60	4.42 ± 2.22	7.45 ± 4.95
Total SFA	34.97 ± 3.77	40.37 ± 5.42	35.02 ± 7.65
C14:1	0.34 ± 0.07	0.17 ± 0.04	0.22 ± 0.04
C16:1 Palmitoleic	5.02 ± 0.83	3.98 ± 0.61	3.83 ± 0.55
C18:1 Oleic	36.16 ± 0.38	33.93 ± 0.46	37.87 ± 0.66
Other MUFA	1.08 ± 0.19	0.94 ± 0.15	0.75 ± 0.10
Total MUFA	42.61 ± 1.50	39.01 ± 1.26	42.67 ± 1.35
C18:2 Linoleic	8.66 ± 1.24	8.30 ± 1.43	9.50 ± 1.65
Other n-6 PUFA	5.94 ± 1.43	5.63 ± 1.17	5.79 ± 0.94
Total n-3 PUFA	1.17 ± 0.28	1.06 ± 0.30	0.90 ± 0.18
Total PUFA	22.40 ± 4.15	20.60 ± 3.92	22.28 ± 3.56
CPFAs ^1^	0.03 ± 0.01 ^a^	0.01 ± 0.01 ^b^	0.03 ± 0.01 ^a^

^1^ CPFAs: cyclopropane fatty acids; it refers to the sum of two isomers, namely DHSA and lactobacillic acid, according to Caligiani et al. [20]. Values are expressed as means ± SEMs (*n* = 10). Values in a row with different letters (when reported) are significantly different (*p* < 0.05; one-way ANOVA with repeated measures, post-hoc Bonferroni). SFA: Saturated Fatty Acids; MUFA: Monounsaturated Fatty Acids; PUFA: Polyunsaturated Fatty Acids.
